# Differential evolutionary conservation of motif modes in the yeast protein interaction network

**DOI:** 10.1186/1471-2164-7-89

**Published:** 2006-04-25

**Authors:** Wei-Po Lee, Bing-Chiang Jeng, Tun-Wen Pai, Chin-Pei Tsai, Chang-Yung Yu, Wen-Shyong Tzou

**Affiliations:** 1 Department of Information Management, National University of Kaohsiung, Taiwan; 2 Department of Information Management, National Sun Yat-sen University, Taiwan; 3 Department of Computer Science, National Taiwan Ocean University, Taiwan; 4 Department of Applied Mathematics, Providence University, Taiwan; 5 Institute of Bioscience and Biotechnology, National Taiwan Ocean University, Taiwan; 6 Center for Marine Bioscience and Biotechnology, National Taiwan Ocean University, Taiwan

## Abstract

**Background:**

The importance of a network motif (a recurring interconnected pattern of special topology which is over-represented in a biological network) lies in its position in the hierarchy between the protein molecule and the module in a protein-protein interaction network. Until now, however, the methods available have greatly restricted the scope of research. While they have focused on the analysis in the resolution of a motif topology, they have not been able to distinguish particular motifs of the same topology in a protein-protein interaction network.

**Results:**

We have been able to assign the molecular function annotations of Gene Ontology to each protein in the protein-protein interactions of *Saccharomyces cerevisiae*. For various motif topologies, we have developed an algorithm, enabling us to unveil one million "motif modes", each of which features a unique topological combination of molecular functions. To our surprise, the conservation ratio, i.e., the extent of the evolutionary constraints upon the motif modes of the same motif topology, varies significantly, clearly indicative of distinct differences in the evolutionary constraints upon motifs of the same motif topology. Equally important, for all motif modes, we have found a power-law distribution of the motif counts on each motif mode. We postulate that motif modes may very well represent the evolutionary-conserved topological units of a protein interaction network.

**Conclusion:**

For the first time, the motifs of a protein interaction network have been investigated beyond the scope of motif topology. The motif modes determined in this study have not only enabled us to differentiate among different evolutionary constraints on motifs of the same topology but have also opened up new avenues through which protein interaction networks can be analyzed.

## Background

In the post-genomic era, one major goal of functional genomics has been to identify and analyze molecular interactions in a cellular context to better understand the mechanisms according to which biological molecules interact and function. A protein-protein interaction (PPI) is a specific type of molecular interaction that plays the central role in relaying signals, in building molecular machines, in engaging in enzyme reactions and in decision-making vis-à-vis multiple biological processes. Advancements in PPI-detection technology have unquestionably led to the rapid accumulation of PPI data [1-5]. To accurately conceptualize a PPI by determining its precise description is to immediately allow for the utilization of the various tools currently accessible in network science [6]. A PPI network, for instance, has been found to have a scale-free structure; i.e., the link count (interactions) of a protein (node) follows a broad-tailed distribution that is approximated as a power-law,*P(k)~k^-γ^*, where *k *is the link count and γ is the degree exponent [6-9]. A network diameter, defined as the average minimal path between two nodes, is reportedly small (~6–7) in a PPI network [9,10].

The contribution made by research on network motifs, where a network motif is the specific topology of a combination of nodes that occurs repeatedly at different positions in that network, is evidenced in the results obtained from the initial search for cause of the topological properties. As the first step, one of the properties of a PPI network, the scale-free feature, was thoroughly examined [11,12]. At issue was whether the selection force is the cause of one of the scale-free network features: a random mutation does not harm a network, as a whole, but can cause it to collapse, but only with a deliberate attack on the hubs (nodes that contain many immediate neighbors) [13]. It was, therefore, reasonably inferred and later clearly observed that hubs are more likely to evolve at a slower rate than non-hub nodes [14,15]. However, different conclusions have been drawn regarding the mutation rate of a hub [12,16]. In addition to this, recent research into gene regulatory networks in yeast has uncovered both a power-law distribution for the number of regulated genes per regulating protein [17] and, at the same time, has yielded invaluable information concerning the presence of typical patterns of motifs. Feed forward motifs, single-input motifs and dense overlapping regulons [18-21] reportedly occur in the gene regulatory network (transcription factor vs. the regulated gene) of *Saccharomyces cerevisiae *and *Escherichia coli *with greater frequency than they would based on chance alone. Network motifs have also been analyzed in the PPI network of *Saccharomyces cerevisiae*, and from this, it has been concluded that motif topology is correlated with the conservation of motif proteins; besides this, it has been inferred that motifs probably represent the evolutionary-conserved topological units of cellular networks [22].

True that the representation of a gene regulatory network where the transcriptional regulator is considered the master and the regulated gene the slave [18,19,22-24] is appropriate, but such a dichotomous (master-slave) representation cannot be used for a PPI since proteins have multiple functions. In this study, we labeled the protein nodes on the basis of their functional attributes of Gene Ontology (GO) [25]. We then pursued any recurring patterns of the functional attributes of protein interactions. With this new representation of a PPI network and by categorizing the repertoire of network motifs of the same topology into "motif modes," with each motif mode featuring a special topological combination of molecular functions, we have been able to move one step ahead of what was accomplished by Barabási's group [22]. This, in turn, led us to the finding that the evolutionary constraints on the motifs of the same topology are certainly not the same when considering their functional attributes; on the contrary, they vary a great deal.

## Results

### Motif topology

**Figure 1:**
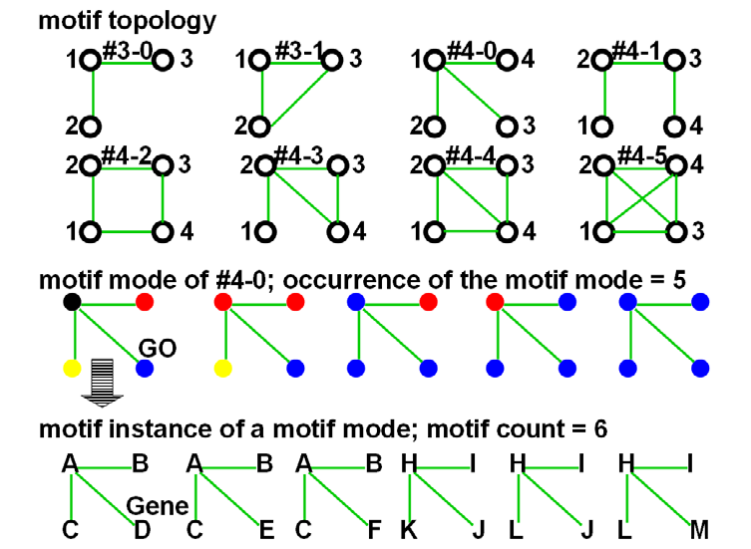
**Motif topology, motif mode, motif instances and motif count. **Eight motif topologies of three-node and four-node motifs are labeled as indicated #3–0, #3–1, #4–0, #4–1, #4–2, #4–3, #4–4 and #4–5. Motif modes of a motif topology (e.g. #4–0) are shown, each colored node indicating GO terms. Motif instances are examples of motifs derived from one of the motif modes, with each node given a gene name.

**Figure 2:**
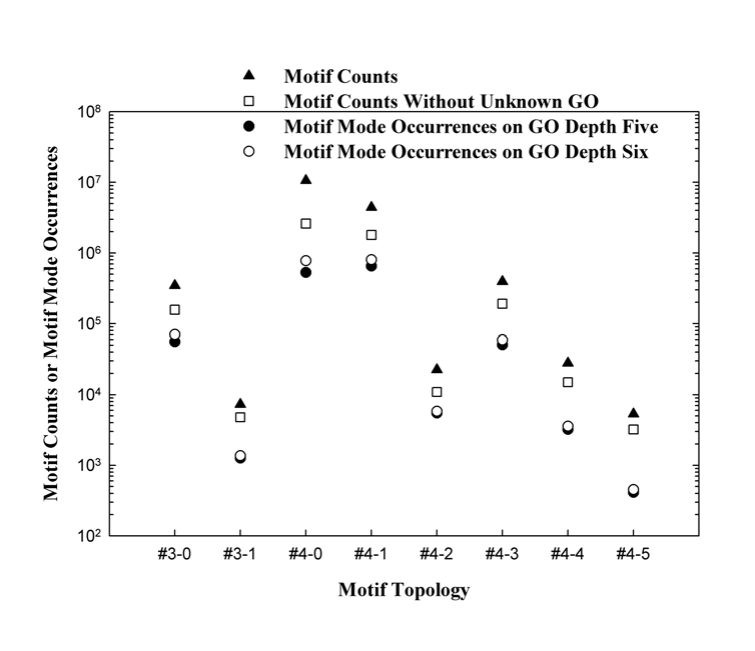
**Frequencies of motif topologies and motif modes in the yeast PPI network. **Motif counts (in filled triangles and open squares) of eight motif topologies and occurrences (in filled and open circles) of the motif modes of each motif topology on a semi-log plot. If the motifs containing the protein nodes of which the GO annotations are GO:0005554 (function unknown) or have no GO annotation, the motifs were disregarded and plotted in open squares. Occurrences of motif modes are indicated as filled and open circles when the GO terms at depths five and six were employed, respectively.

The PPI data for *Saccharomyces cerevisiae *contain a total of 15,129 physical interactions formed by 4,738 proteins (see Methods). We analyzed the occurrence of eight types of three-node and four-node motifs, referred to hereafter as the "motif topology" (Figure 1). The motif counts (i.e., the number of instances) of eight motif topologies varies, from 5.3 thousand (four-node motif topology 5, #4–5 hereafter) to an overwhelming 10.6 million (four-node motif topology 0, #4–0 hereafter) (Figure 2; see additional file 1-Table S1).

### Motif mode

**Figure 3:**
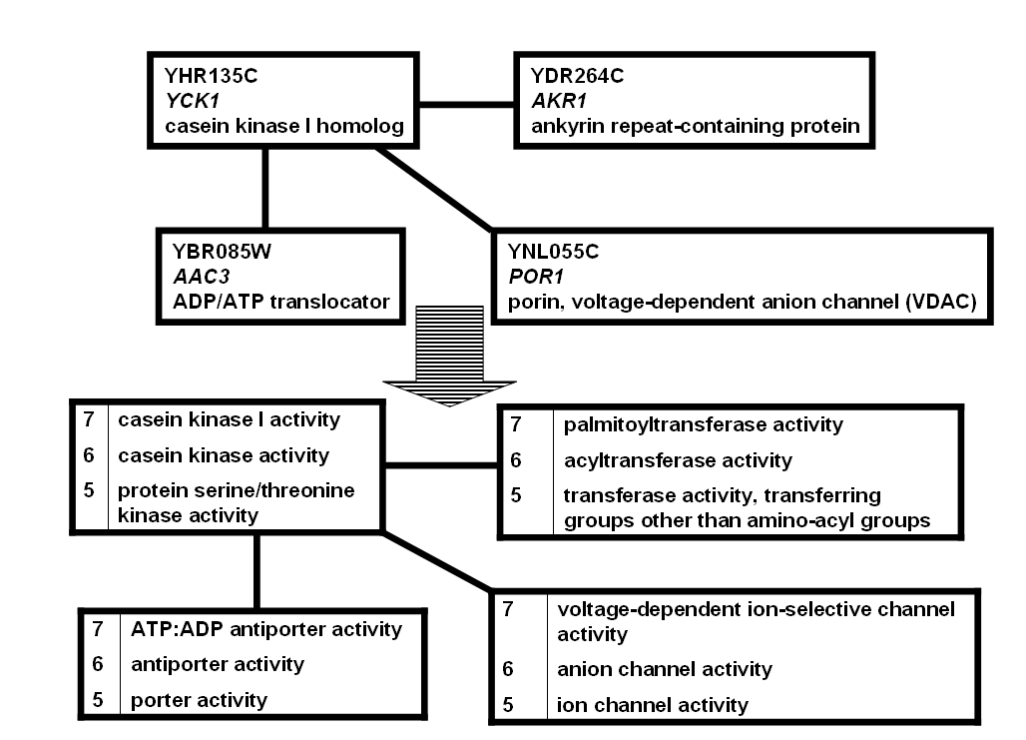
**Annotation of GO terms at different depths on the constituents of a typical #4–0 motif. **A typical #4–0 motif. For each protein, the open reading frame name, the gene name and the full name are provided. GO terms for each node are provided at three GO depths (five, six and seven). All nodes in the PPI network are labeled with GO terms for the discovery of the motif mode.

Since we focused on the recurring patterns of the functional attributes of protein interactions, we used the molecular function ontology. On these grounds, we used the molecular function ontology (hereafter GO terms) to annotate each node of a PPI network (Figure 3).

**Figure 4:**
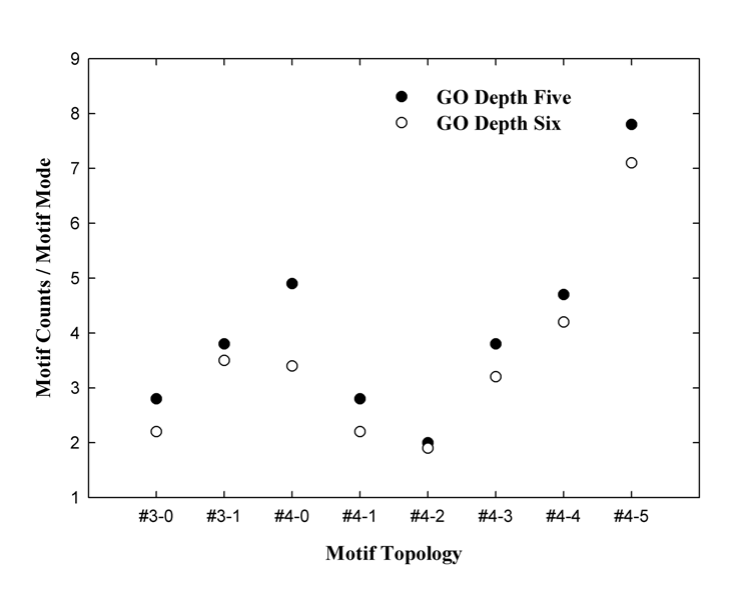
**Average motif counts per motif mode of each motif topology in the yeast PPI network. **Average motif counts per motif mode for each motif topology are indicated as filled and open circles when the GO terms at depths five and six were employed, respectively.

We developed a new algorithm (see Methods) to categorize network motifs into "motif modes" on the basis of the topology of the GO terms annotated for motif nodes (Figure 1). Here a "motif mode" is defined as a special combination of GO terms in a motif. In fact, a motif mode illustrates a grouping of network motifs that contain the same topological combination of functional attributes of proteins (Figure 1). When we employed the GO terms at depths five and six, we found a total of nearly 1.3 and 1.7 million motif modes, respectively (Figure 2; see additional file 1-Table S1). In general, the higher motif count a particular motif topology has, the more motif modes there are in that motif topology (correlation coefficient = 0.96, *p *< 0.001). However, #4–5 has the largest counts per motif (on average, around eight motif counts per motif mode; Figure 4; see additional file 1-Table S1).

**Figure 5:**
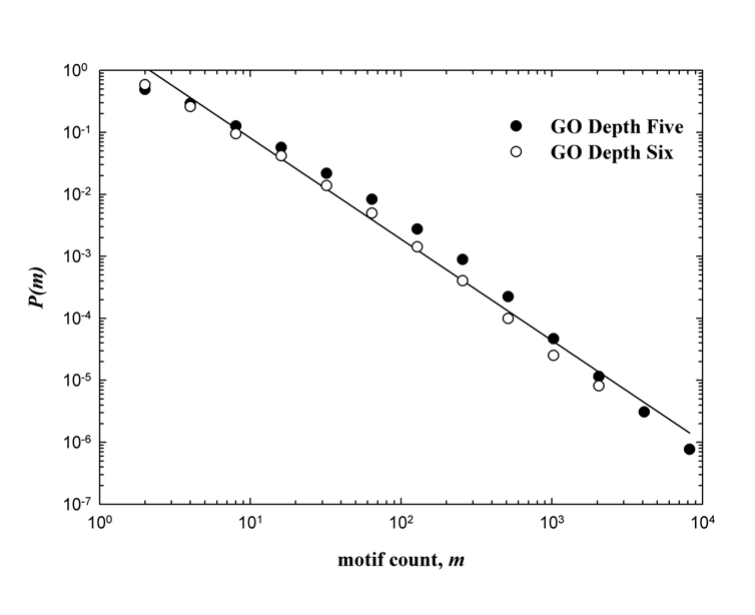
**Distribution of motif counts for motif modes in the yeast PPI network. **Distribution of motif counts (logarithmic binning) for motif modes shown on a log-log plot are marked as filled and open circles when the GO terms at depths five and six were employed, respectively. The solid line indicates a fit to *P(m)~m^-φ ^*where *m *is the motif count and φ is the degree exponent ~1.6.

Noteworthy too is that the motif count for each motif mode can vary considerably among a million motif modes. We calculated the distribution of the motif count for each motif mode based on the logarithmic binning. When we employed the GO terms at depths five and six, nearly 49% and 58% of the motif modes respectively occur less than 2 times in a PPI network (Figure 5; see additional file 1-Table S2). It is particularly interesting that, for all motif modes, there is a power-law distribution of the motif counts, *P(m)~m*^*-φ*^, where *m *is the motif count and *φ *is the degree exponent ~1.6 (R square = 0.99, *p *< 0.001) (Figure 5). In fact, quite a few motif modes contain more than one hundred motif counts (1,577 and 1,449 motif modes when we employed the GO terms at depths five and six, respectively).

In that motif modes allow for an analysis that extends beyond motif topology, we were able to look at the properties of motifs of the same topology at a higher resolution than had ever been done before [22].

### Annotation transfer within a protein interaction network

Since we were interested in finding the evolutionary trend of the proteins contained in a motif mode, we built an orthology gene list among the genes of the six organisms (*Saccharomyces cerevisiae*, *Arabidopsis thaliana*, *Caenorhabditis elegans*, *Drosophila melanogaster*, *Mus musculus*, and *Homo sapiens*). When the orthology gene list was used to compute the evolutionary constraint of motifs of different motif topology and motif modes, it was assumed that orthologous proteins also interact with each other. It has been shown that the reliability of the annotation of protein interactions from one organism to another depends on the level of sequence identity for the two pairs of interacting proteins [26]. The joint E-value (the geometric means of the BLAST E-values for the two pairs of interacting proteins) smaller than <10^-70 ^was used as the threshold to evaluate whether such a transfer of the annotation of interactions is reliable [26]. In order to investigate the validity of the orthology gene list by which the interaction annotations were transferred between organisms, we conducted a sequence comparison between orthologs of yeast and five other species. We found 61% of the orthologous protein pairs have an E-value lower than 10^-70 ^(see additional file 2-Sup2.doc, Figure S1). About 40% of the annotation transfers are less reliable; just the same, we have to emphasize that the orthology approach still remains important in terms of decoding many important biological phenomena [22,27].

### Different evolutionary constraints on the motifs of the same topology

**Figure 6:**
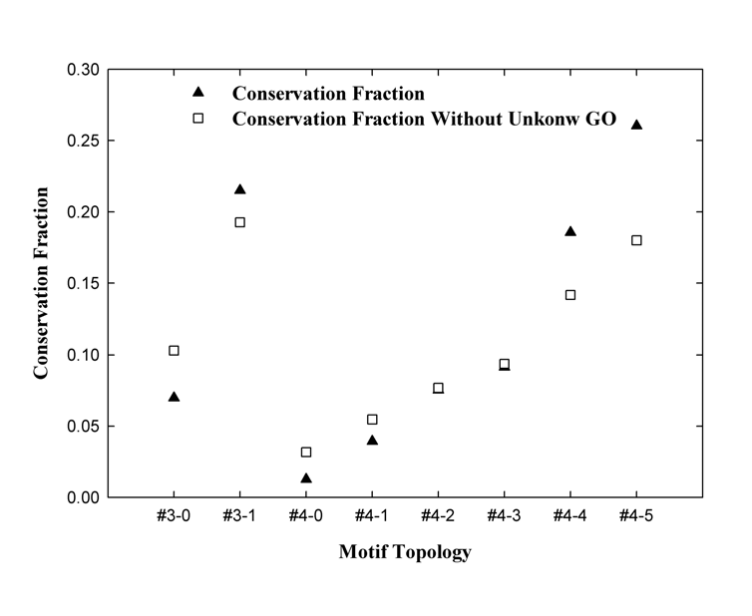
**Average conservation fraction of each motif topology in the yeast PPI network. **Average conservation fraction (in filled triangles and open squares) of each motif topology. If the motifs containing the protein nodes of which the GO annotations are GO:0005554 (function unknown) or have no GO annotation, the motifs were disregarded and plotted in open squares.

**Figure 7:**
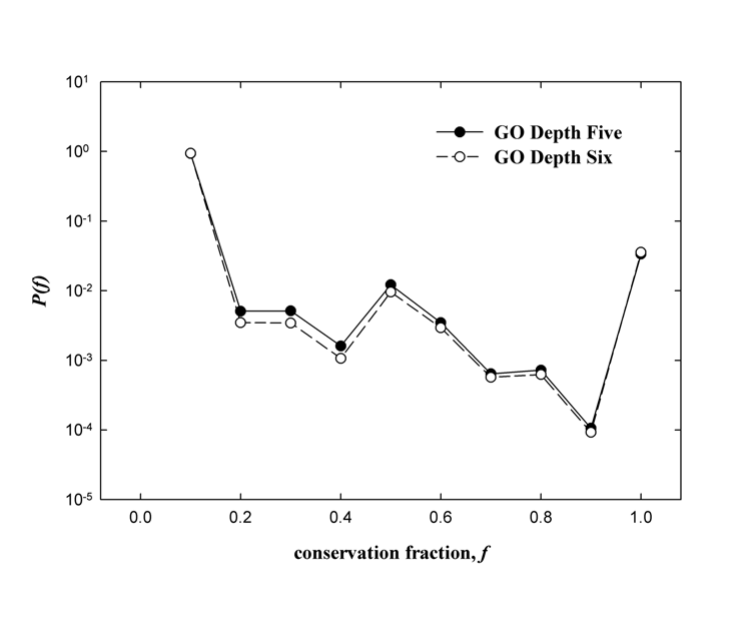
**Distribution of the conservation fraction of motif modes in the yeast PPI network. **Distribution of the conservation fraction of motif modes shown on a semi-log plot are marked as filled and open circles when the GO terms at depths five and six were employed, respectively.

To discover the evolutionary trend of the proteins contained in a motif mode, we computed the "conservation fraction" for each motif topology. The "conservation fraction" is the counts of the evolutionary fully conserved motif divided by total motif counts. Motifs #3–1, #4–4, and #4–5 have higher conservation fractions (0.19 ~0.26) than do the others (Figure 6; see additional file 1-Table S3). Important to note, these values are very similar to those reported earlier (0.22 vs. 0.21 for #3–1, 0.19 vs. 0.19 for #4–4, 0.26 vs. 0.33 for #4–5) [22]. We also calculated the distribution of the conservation fractions of each motif mode using 0.1 as the bin. When we employed the GO terms at depths five and six, the conservation fraction of nearly 93% and 94% of all motif modes respectively was less than 0.1 (Figure 7; see additional file 1-Table S4). This is due to the fact that the majority of motif counts occur for #4–0 and #4–1 (Figure 2), with the average conservation fraction of ~0.05 (Figure 6). However, we also noted that the conservation fraction for 3.4% and 3.6% of motif modes, when we employed the GO terms at depths five and six respectively, reaches 1.0. The general trend stated above still holds if the distribution is shown on the basis of each motif topology (see additional file 2-Sup2.doc, Figure S2).

**Figure 8:**
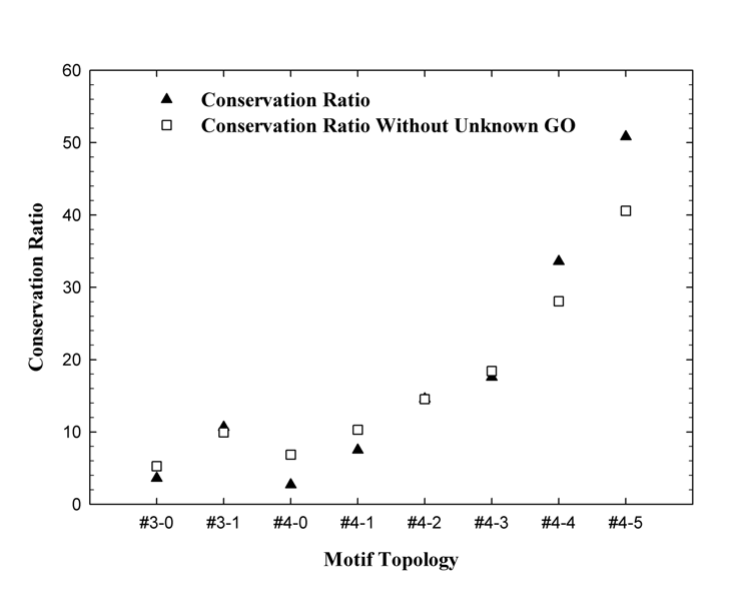
**Average conservation ratio of each motif topology in the yeast PPI network. **Average conservation ratio (in filled triangles and open squares) of each motif topology. If the motifs containing the protein nodes of which the GO annotations are GO:0005554 (function unknown) or have no GO annotation, the motifs were disregarded and plotted in open squares.

We defined the "conservation ratio", a degree of evolutionary constraint, as the value of the conservation fraction stated above divided by the same value but computed after the random assignment of the orthology data to the proteins (see Methods). It is apparent that the more connected to each other the motif nodes are, the higher is the conservation ratio (e.g., #4–5 > #4–4 > #4–3 > #4–2 > #4–1 > #4–0; #3–1 > #3–0) (correlation coefficient = 0.71, *p *= 0.05; Figure 8; see additional file 1-Table S3). This observation has been previously reported [22].

**Figure 9:**
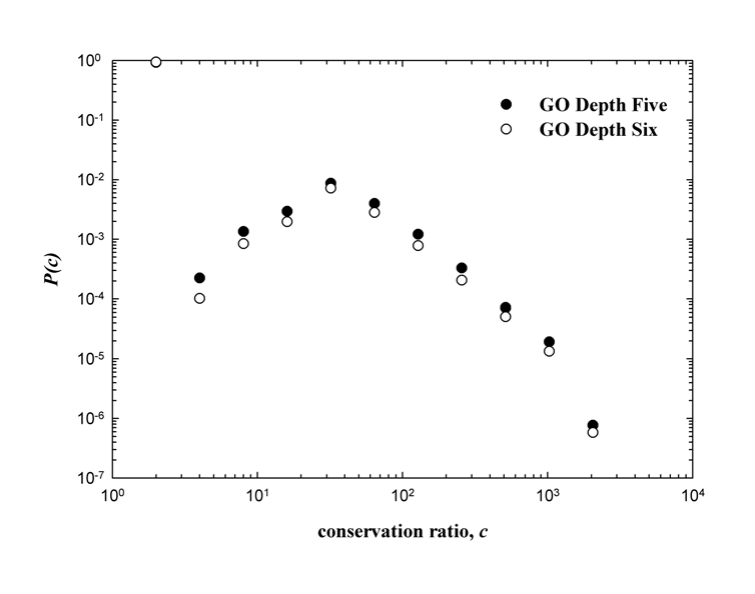
**Distribution of the conservation ratio of the motif modes in the yeast PPI network. **Distribution of the conservation ratio of motif modes shown on a log-log plot are marked as filled and open circles when the GO terms at depths five and six were employed, respectively.

To determine if the conservation ratio is the same for motif modes of the same topology, we calculated the conservation ratio for one million motif modes. We calculated the distribution of the conservation ratio for each motif mode based on the logarithmic binning. What we found is that when we employed the GO terms at depths five and six, the conservation ratio for more than 93% and 94% of the motif modes is respectively lower than 2. Surprisingly, we also noted that the conservation ratio for 3,510 (GO terms at depth five) and 3,096 (GO terms at depth six) motif modes exceeds 50, which is approximately the magnitude of the highest ratio observed for motif #4–5 (Figures 8, 9; see additional file 1-Table S5). Support for the notion that the evolutionary constraints on motifs of the same topology are not the same gains considerable ground, and this suggests the presence of differential evolutionary constraints upon motif modes of the same motif topology. We can fit the distribution of the conservation ratio for all motif modes on a logarithmic scale by employing a quadratic function if the value of the first bin (0~2) is not taken into account (R square = 0.97, *p *< 0.001). The above observations still hold if the distribution is shown on the basis of each motif topology (see additional file 2-Sup2.doc, Figure S3).

### A motif mode by chance alone?

As a unique combination of molecular function descriptions of GO, a motif mode could possibly be the result of a special classification that occurs by chance. We randomized the GO annotations (molecular function) on the nodes and re-calculated three features of each motif mode: the motif counts, the conservation fraction and the conservation ratio. We found a significant difference in the motif counts and the conservation ratio of the motif modes with and without the randomization of the GO annotations (*p *<= 0.0001; see additional file 1-Table S6). Therefore, the motif modes categorized by the current GO annotations and the properties derived from the motif modes (e.g., the level of differential evolutionary conservation) do indeed bear a greater significance than would normally be expected by chance alone.

## Discussion

We used nearly 400 and 650 GO molecular function descriptions at different depths to annotate nearly 5,000 protein nodes in the *Saccharomyces cerevisiae *PPI network. We employed a motif mode to represent any probable combination of GO annotations with a three-node and four-node topology. To this effect, we collected all of the existing million motif modes and examined the level of evolutionary constraints on their motif constituents (the conservation ratio). We found two interesting distributions of the properties of the motif modes. The first is the distribution of motif counts a motif mode consists of, *P(m)~m*^*-φ*^, where *m *is the motif count and φ is the degree exponent ~1.6 (Figure 5). This observation may be related to a recent finding that, in a complex network, the large-scale topological organization (characterized by the degree exponents of the scale-free and hierarchical modularity) and the variable counts of the different motif topologies can define each other [28,29]. Whether there is a direct correlation between the large-scale topological organization and the motif mode-motif count dependence has yet to be determined. The second is the distribution that characterizes the dependence of the number of motif modes on the conservation ratio of that motif mode (Figure 9, Figure S3). Reportedly, the more interconnected the nodes of a motif are with each other, the more conserved the protein constituents of the motif are [22]. If a motif, rather than a single protein (e.g. hub), represents evolutionary-conserved topological units in the tapestry of a PPI network [22], our study further shows that motifs belonging to different motif modes of the same topology are not under the same level of evolutionary constraints. Fewer motif modes are under higher evolutionary constraints, and the level of differences spans the order of three.

### Differences between this and previous studies

The use of GO annotations has provided us with increased insight into protein interactions through the coloring of protein nodes in the interaction map [30]. In this study, we categorized motifs on the basis of the topological combinations of GO terms (molecular function), and this has resulted in our discovery of motif modes. It should not be overlooked that motifs in biological networks have previously been analyzed [18,22]. The Alon group, for example, developed Mfinder to calculate the motif counts in gene-regulatory networks [18]. Our algorithm is capable not only of conducting motif counts but also of managing the grouping of motif modes based on GO terms; as mentioned earlier, this analysis, in fact, reaches a higher resolution than any other reported before. We recorded the protein identities and GO terms of each motif using our algorithm for the computation of the conservation ratio. We recorded all motif instances (five million) of the motif modes (one million), and they can be used in future analyses though this requires large computer memory. For this very reason, we focused on the motif modes of three nodes and four nodes in this study.

Until now, the evolutionary constraints have only been reported on the level of motif topology, and this by computing the evolutionary constraints of all motifs of the same motif topology [22]. In this study, for the first time, we have been able to distinguish the evolutionary constraints of different motifs of the same topology. A motif topology contains many motif modes which are under different levels of evolutionary constraints. In proposing that motif modes may represent the evolutionary-conserved topological units of a protein interaction network, we have clearly progressed one step farther beyond what has been accomplished before.

## Future direction

It is possible to re-examine the motifs and motif modes of PPIs of model organisms without relying on the orthology information. One can take several real PPI networks of different organisms and, for each network, look independently for the motif they contain and use the GO annotations to define motif modes, then compare if the same motif modes are used in these different organisms. Apart from this, it would be most enlightening to investigate the temporal patterns of various motif modes when gene expression data are used. This would be just like a PPI network visualized within the context of cell-cycles [31], hubs categorized as "party hubs," or "date hubs," [32] and topological changes in transcription regulatory networks observed under environmental or physiological conditions [33]. Furthermore, it would be most worthwhile enhancing the precision of module-detection methods [34-40] and developing specialized tools designed to search for motifs with similar functional annotations. This would greatly assist biologists interested in mining protein interaction networks. PathBlast [41], TopNet [42], MAVisto [43], and FANMOD [44] are examples of work in these areas.

## Conclusion

We have reported on using the molecular function vocabulary of GO to annotate a yeast protein interaction network. The motif mode, i.e., the special topological combination of the molecular functions of interacting proteins, was extracted from the yeast protein interaction network and employed in the analysis of the protein interaction network at a higher resolution than ever before. The distribution of the motif counts for all the motif modes follows a scale-free like fashion. The differential evolutionary constraints on the million motif modes are an indication that motif modes may very well represent the evolutionary-conserved topological units of the protein interaction network.

## Methods

### PPI dataset

The yeast (*Saccharomyces cerevisiae*) PPI dataset is from the DIP database [45] (July 2004). There are 15,409 interactions in the original data, and after self-interacting protein interactions were removed, 15,129 interactions composed of 4,738 genes (proteins) remained.

### Gene Ontology associations

The molecular function of the open reading frame (ORF) name of each gene is based on GO annotations (July 8, 2004). Of 4,738 genes, 857 GO terms are used to describe the molecular functions of genes. No GO term (molecular function) can be found for 289 open reading frames, and these account for 499 interactions. An ORF-GO correspondence table is provided in the additional file 1-Table S7.

### Molecular functions of GO terms at different depths

GO terms are organized in structures referred to as 'directed acyclic graphs,' and each term can be traced to different depths in the hierarchies. The molecular function of the GO terms for each protein in our dataset was traced to depths five and six backward to the root. (The root is located at depth 0; "molecular function", GO:0003674, at depth one; "antioxidant activity", GO:0016209, at depth 2). If the GO term for a protein is located at a depth lower than six in the GO tree, the GO term remains unchanged. Our reason for choosing depths five and six is that, based on the statistics, the average depth of the GO terms in our dataset is six (data not shown). The 857 GO terms used to describe a gene's molecular function were then reduced to 398 and 648 terms at depths five and six, respectively.

### Algorithm to detect motif modes

We considered a PPI network as a graph and used the method of an adjacency list to represent the graph. In this representation, we labeled the nodes, and for each node, we created a linked list to record its immediate neighbors. To count the occurrences of each motif mode, we modified and employed the graph-searching algorithm, Depth-First-Search (DFS). To start the search, we used each graph node sequentially as the initial node, with a depth limit corresponding to the number of motif nodes. We performed the DFS-based search in a recursive way and implemented a checking procedure to ensure that we did not repeatedly count any identical structure during the search. When we recognized a special topological structure, we proceeded to map the nodes of that structure to those of the motif based on the links through which the nodes were connected to each other. We then recorded the identity of the nodes of the newly found structure and added them to the set of this specific motif mode.

### Ortholog assignment

We retrieved the orthologous sequence pairs from InParanoid [46] (Version 3.0; updated 15, August 2004) for a bootstrap value of 100% and a score of 1.00 in each cluster. We found 1,247 genes of *Saccharomyces cerevisiae *that have high-confidence orthologs with 1,440 genes from *Arabidopsis thaliana*, 1,286 genes from *Caenorhabditis elegans*, 1,397 genes from *Drosophila melanogaster*, 1,501 genes from *Mus musculus*, and 1,439 genes from *Homo sapiens*. We built an orthology gene list using 1,247 genes of *Saccharomyces cerevisiae *for the protein sequences of the genes orthologous to the five species. Out of 15,129 interactions, 1,247 genes from yeast constitute 2,629 protein interactions.

### Conservation fraction and conservation ratio

We then had to find all the constituents of the evolutionary fully conserved motif in the orthology gene list. For each motif topology and each motif mode, we computed the "conservation fraction", i.e., the counts of the evolutionary fully conserved motifs divided by the total motif counts of the motif topology.

To identify the degree to which a motif is under higher evolutionary constraints than would normally be expected by chance alone, we computed "conservation ratio". We randomly assigned the orthology data to the proteins and re-calculated the conservation fraction, as stated above. We repeated this procedure twenty times and obtained an average value which we termed the "random conservation fraction" (see additional file 1-Table S3). The "conservation ratio" of a motif topology or a motif mode is the ratio of the conservation fraction over the "random conservation fraction" of topology or mode, respectively. If the "random conservation fraction" was zero, we identified the "conservation ratio" as non-determined value (NDV).

## Abbreviations

PPI, Protein-Protein Interaction

GO, Gene Ontology

## Authors' contributions

Wei-Po Lee built the entire algorithm and was charged with its implementation. Bing-Chiang Jeng annotated the protein nodes based on GO. Tun-Wen Pai analyzed the GO terms at different depths. Chin-Pei Tsai and Chang-Yung Yu were responsible for the statistical analysis. Wen-Shyong Tzou conceived the study, participated in its design and coordination and drafted the manuscript.

## Supplementary Material

Additional File 1 Table S1. Motif counts and motif modes of each motif topology. Table S2. Distribution of motif counts (logarithmic binning) for the motif modes of each motif topology. Table S3. Conservation fraction and conservation ratio of each motif topology. Table S4. Distribution of conservation fractions binned in the interval of 0.1 for the motif modes of each motif topology. Table S5. Distribution of conservation ratios (logarithmic binning) for the motif modes of each motif topology. Table S6. Average value and standard deviations of the distribution of the motif counts, conservation fraction and conservation ratios for the motif modes of each motif topology. Table S7. List of the protein-protein interactions (link) and the GO terms (molecular function) of the protein nodes. Click here for file

Additional File 2 Figure S1. Distribution of the joint E value for the amino acid sequence comparison between orthologs of yeast and five other species. Figure S2. Distribution of the conservation fraction of motif modes in the yeast PPI network on the basis of each motif topology. Figure S3. Distribution of the conservation ratio of the motif modes in the yeast PPI network on the basis of each motif topology. Click here for file
