# Supplementary material for: Differential evolutionary conservation of motif modes in the yeast protein interaction network
Source: BMC Genomics. 2006 Apr 25;7:89. doi: 10.1186/1471-2164-7-89 (PMC1501022; doi:10.1186/1471-2164-7-89)
Supplement: Additional File 2 — Figure S1. Distribution of the joint E value for the amino acid sequence comparison between orthologs of yeast and five other species. Figure S2. Distribution of the conservation fraction of motif modes in the yeast PPI network on the basis of each motif topology. Figure S3. Distribution of the conservation ratio of the motif modes in the yeast PPI network on the basis of each motif topology. [file 1471-2164-7-89-S2.pdf]

additional file 2: Filename: Sup2.doc.

Figure S1. Distribution of the joint E value for the amino acid sequence comparison between orthologs of yeast and five other species.

Figure S2. Distribution of the conservation fraction of motif modes in the yeast PPI network on the basis of each motif topology.

Figure S3. Distribution of the conservation ratio of the motif modes in the yeast PPI network on the basis of each motif topology.

Figure S1. Distribution of the joint E value for the amino acid sequence comparison between orthologs of yeast and five other species.

The value “100” in the abscissa indicates the percentage of E value smaller than 10^{-100}.

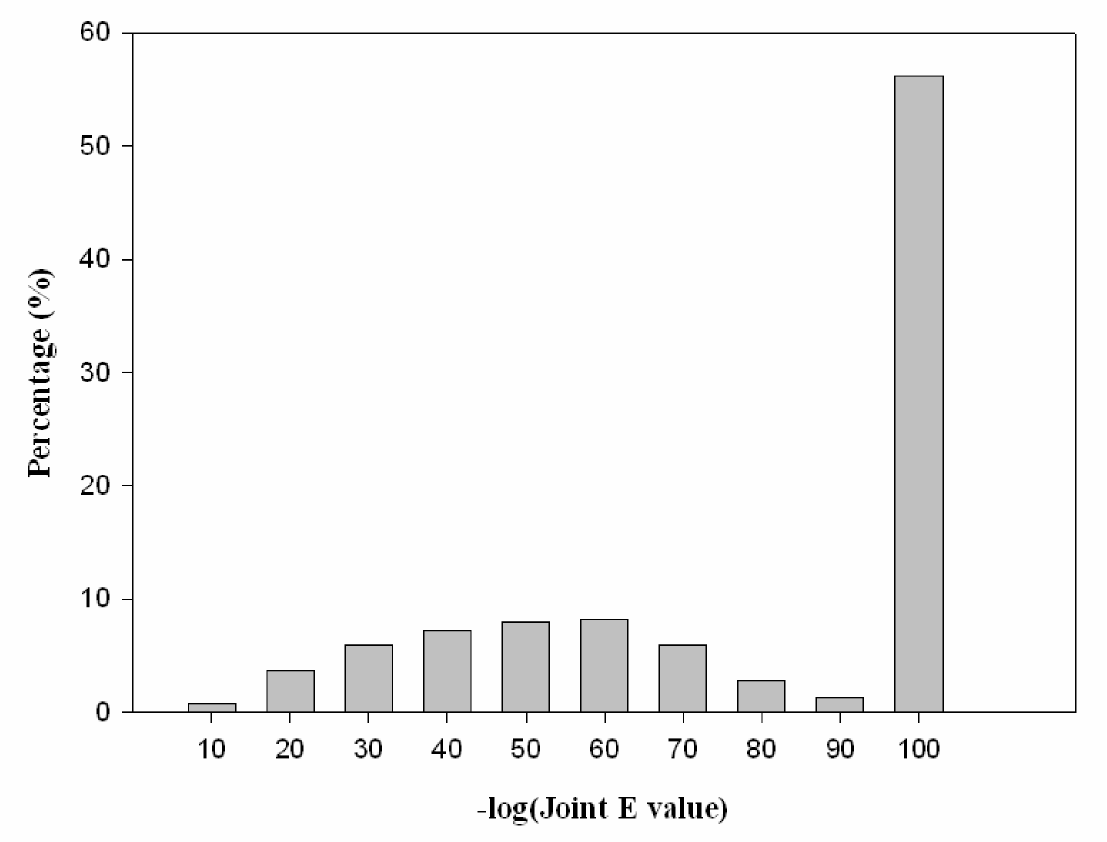

Figure S2. Distribution of the conservation fraction of motif modes in the yeast PPI network on the basis of each motif topology.

Distribution of the conservation fraction of motif modes shown on a semi-log plot are marked as filled and open circles when the GO terms at depths five and six were employed, respectively.

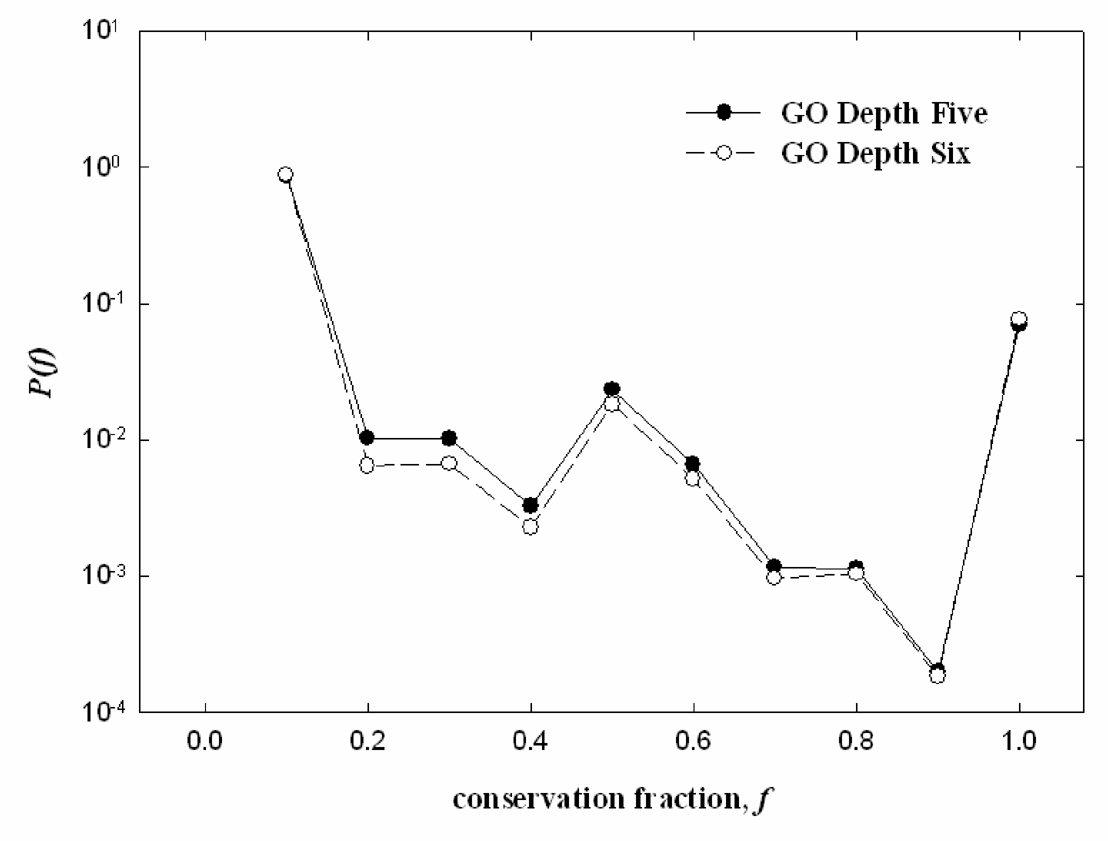

Motif topology: #3-0

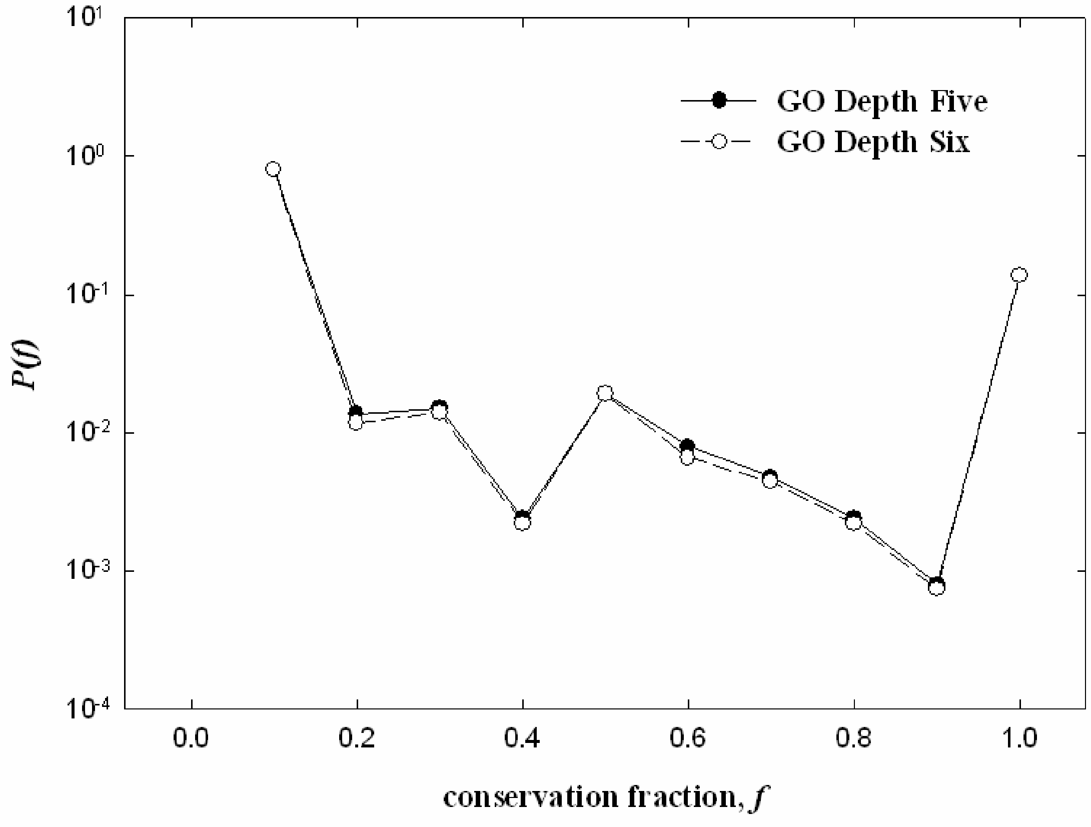

Motif topology: #3-1

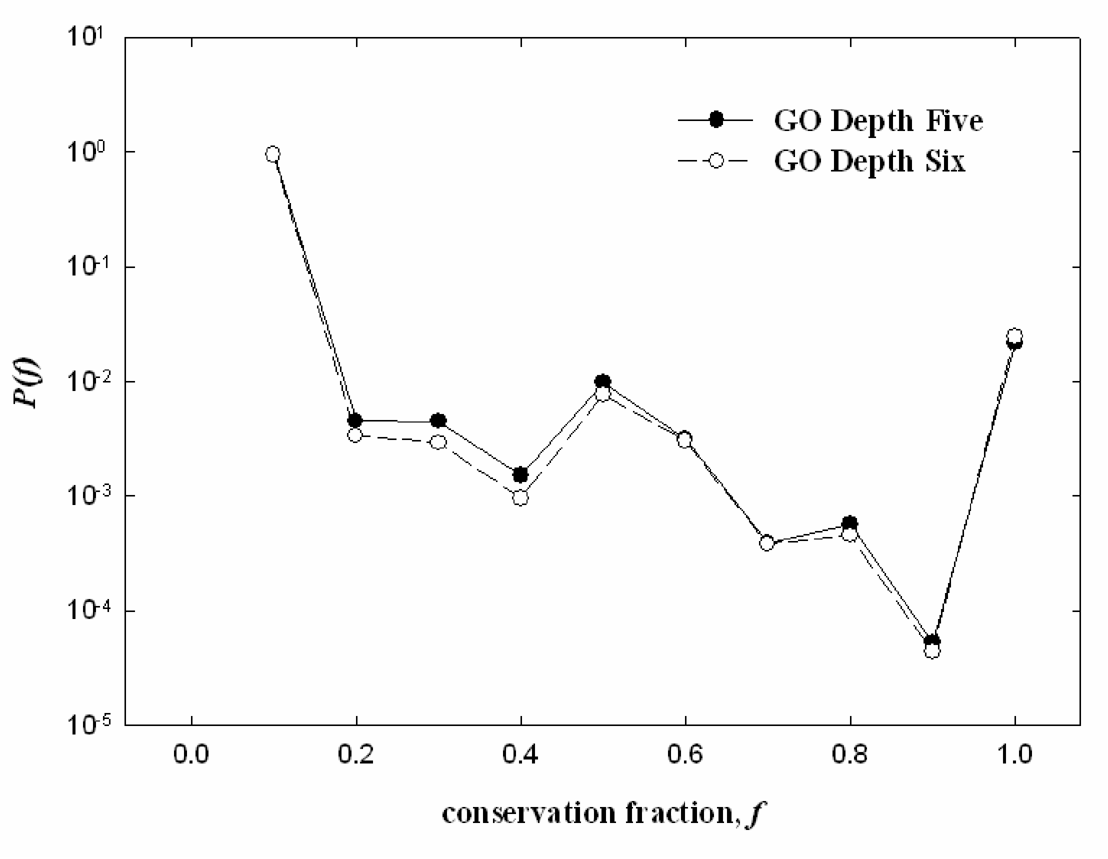

Motif topology: #4-0

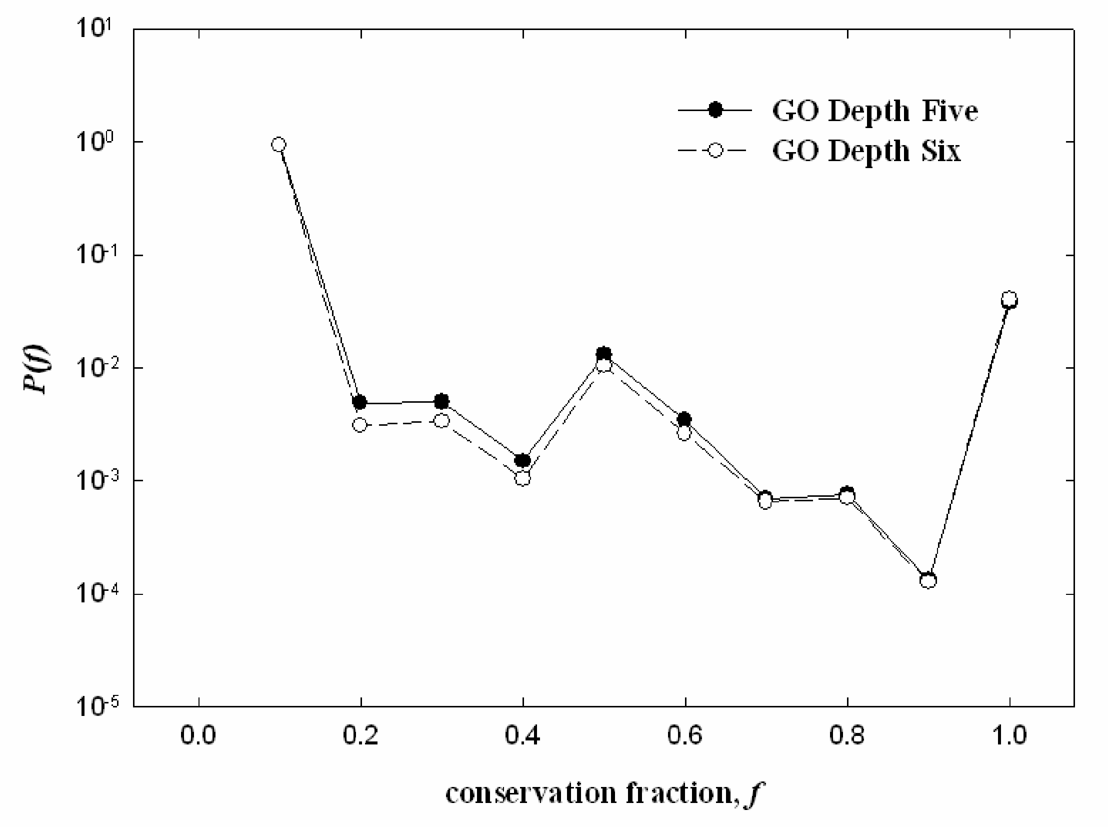

Motif topology: #4-1

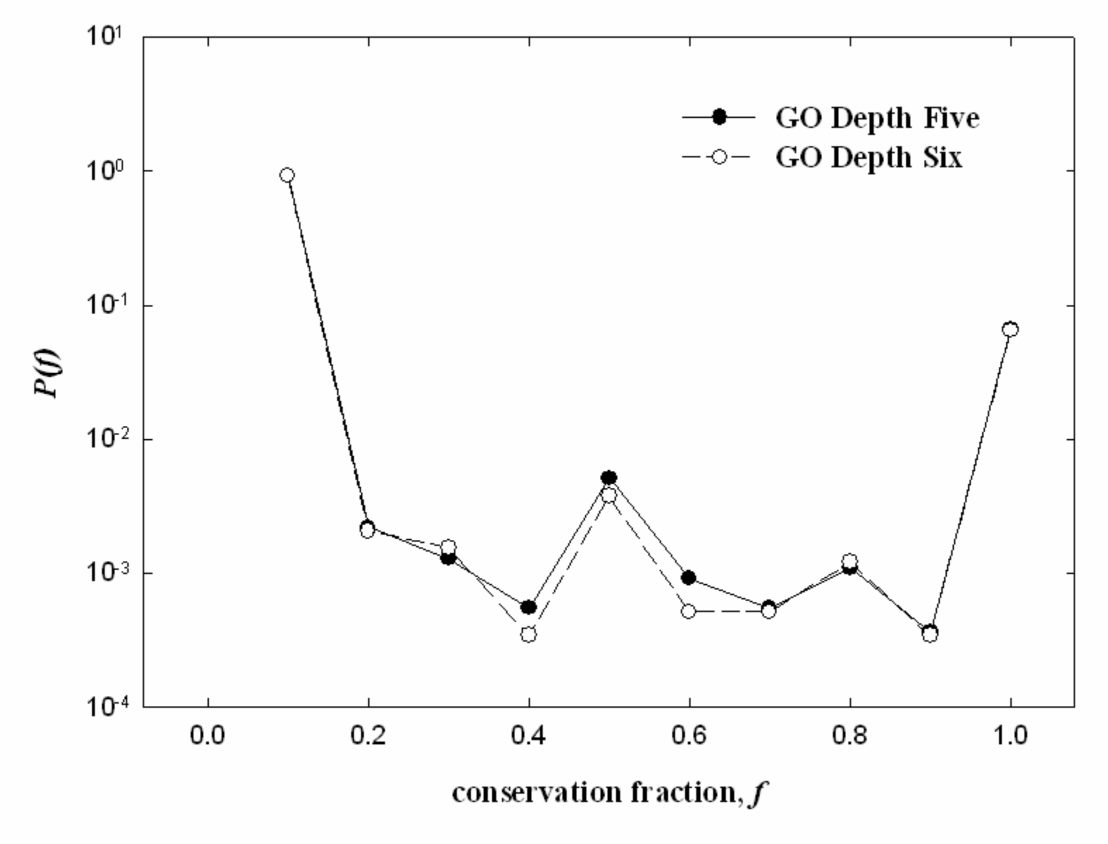

Motif topology: #4-2

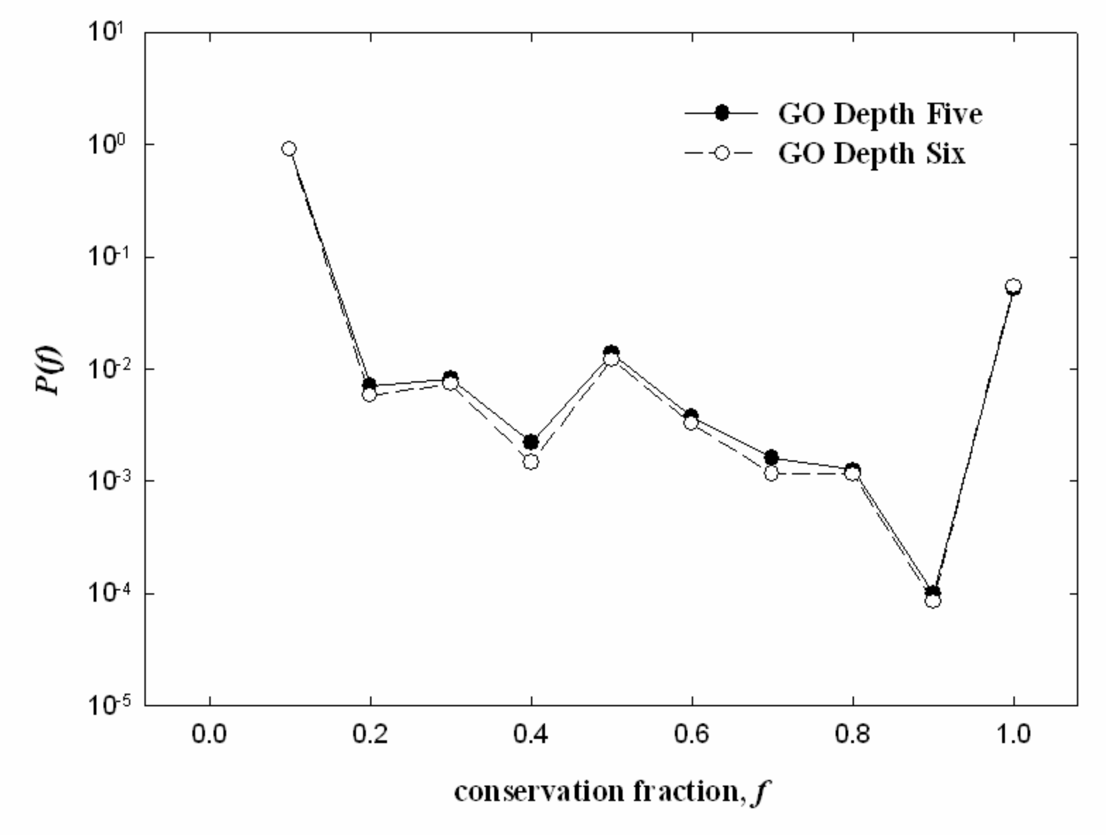

Motif topology: #4-3

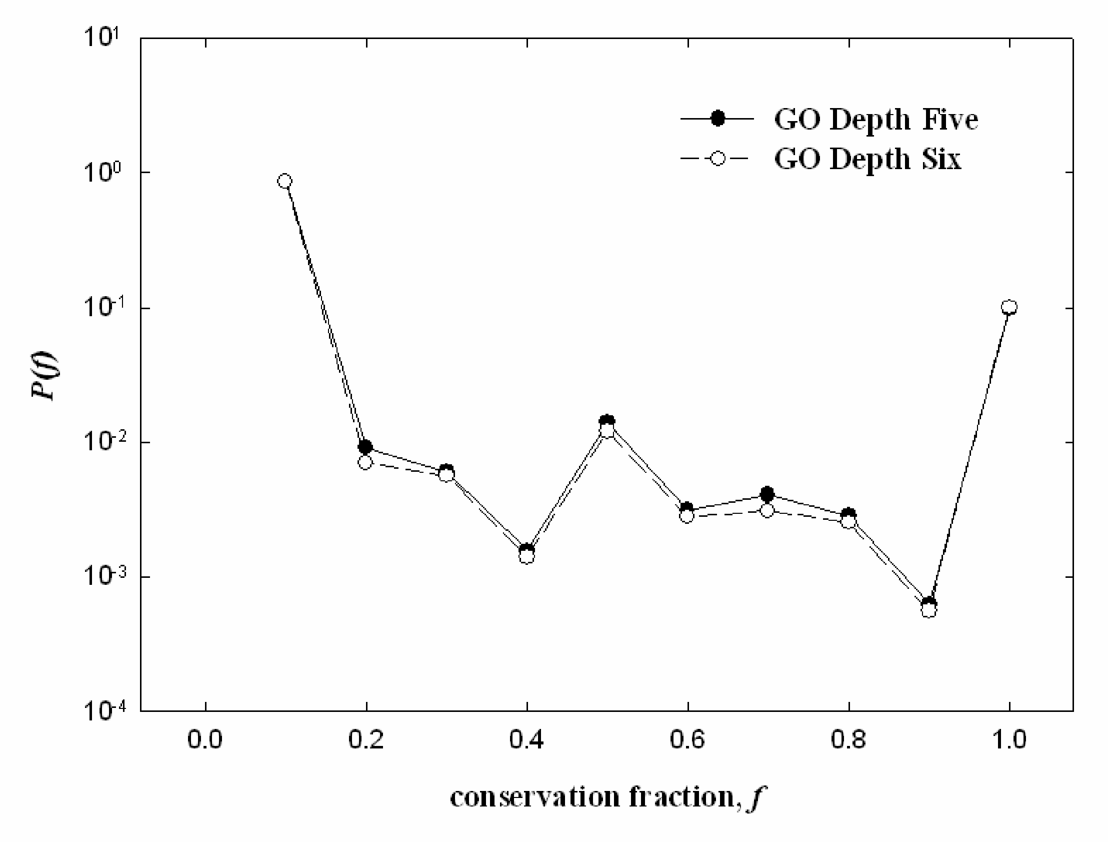

Motif topology: #4-4

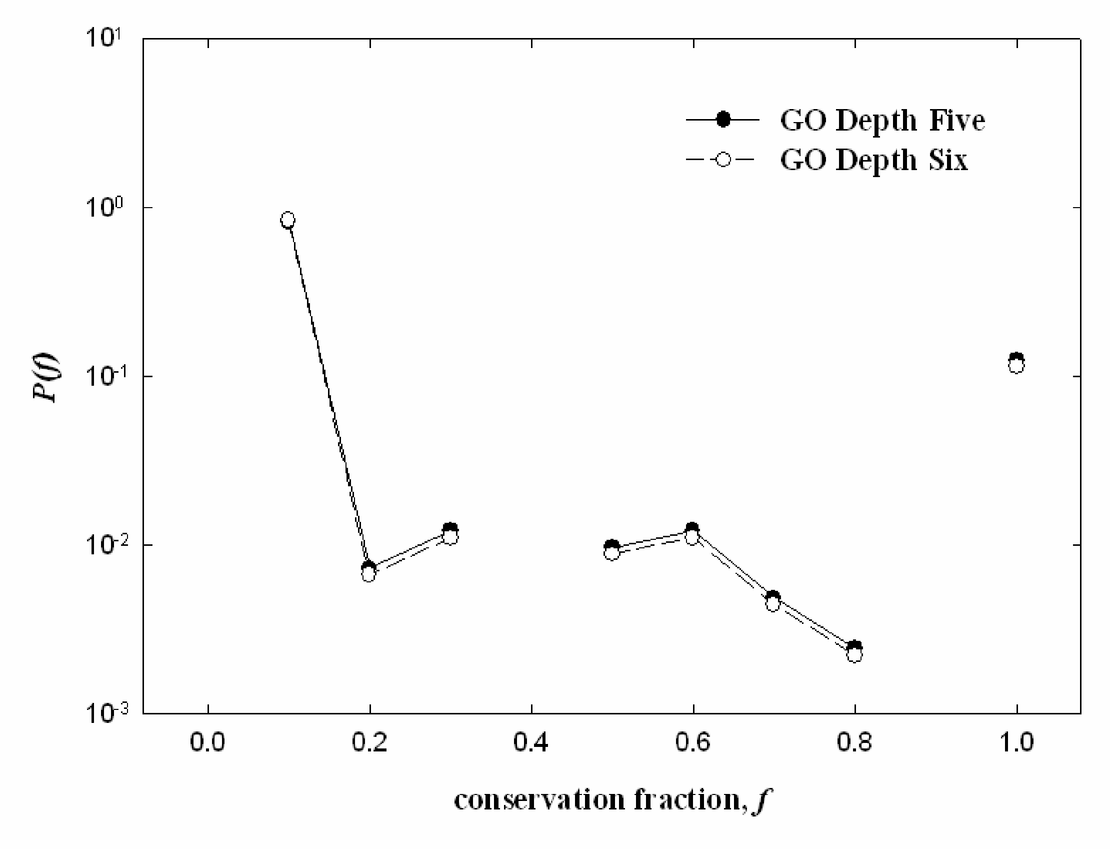

Motif topology: #4-5

Figure S3. Distribution of the conservation ratio of the motif modes in the yeast PPI network on the basis of each motif topology.

Distribution of the conservation ratio of motif modes shown on a log-log plot are marked as filled and open circles when the GO terms at depths five and six were employed, respectively.

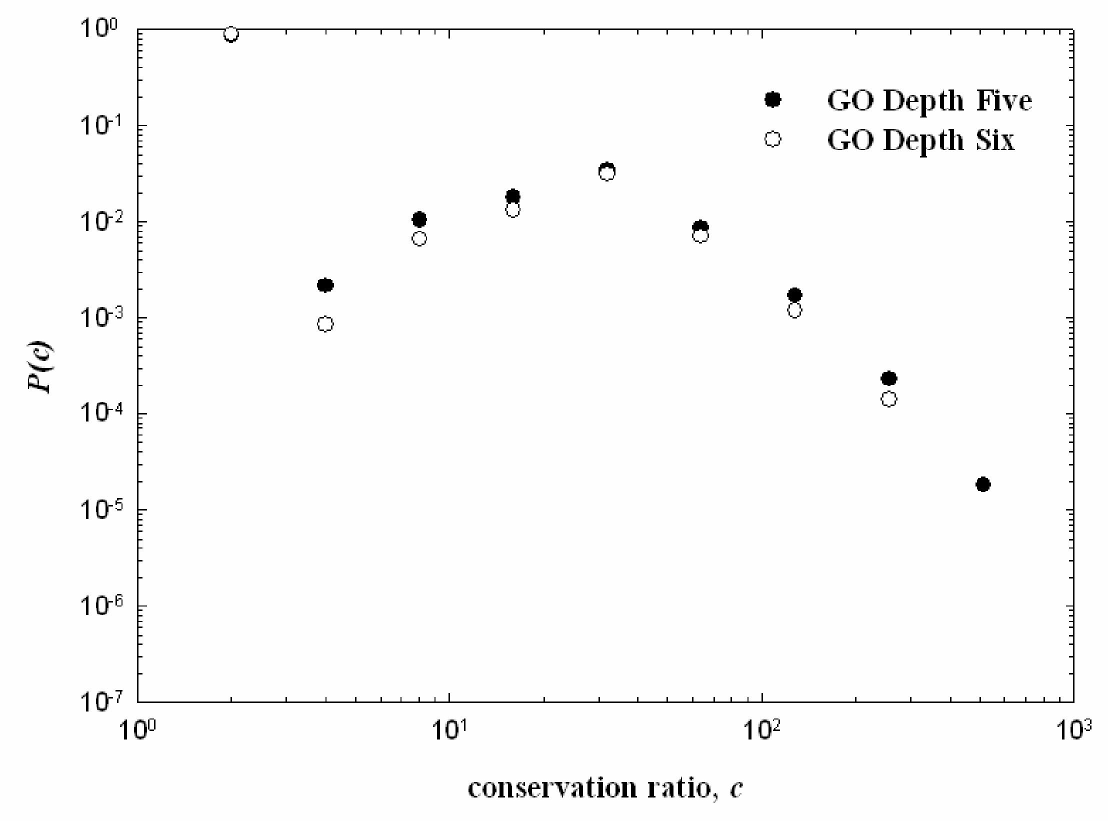

Motif topology: #3-0

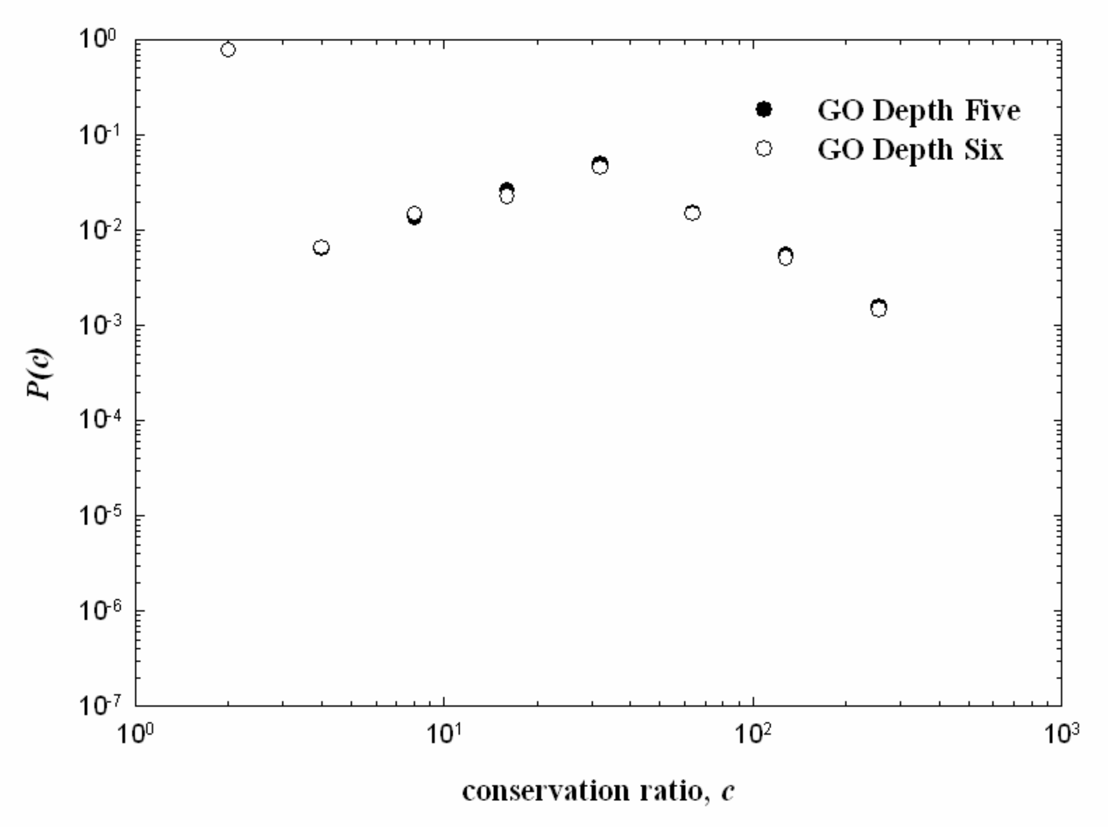

Motif topology: #3-1

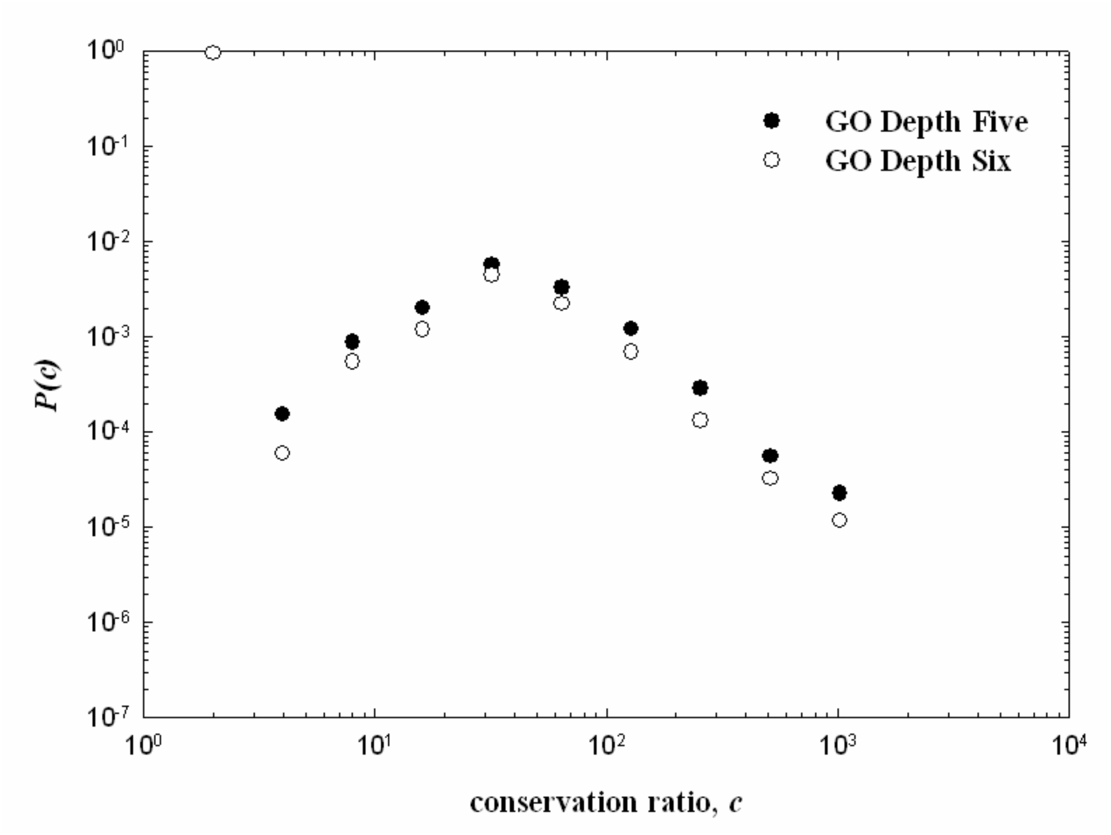

Motif topology: #4-0

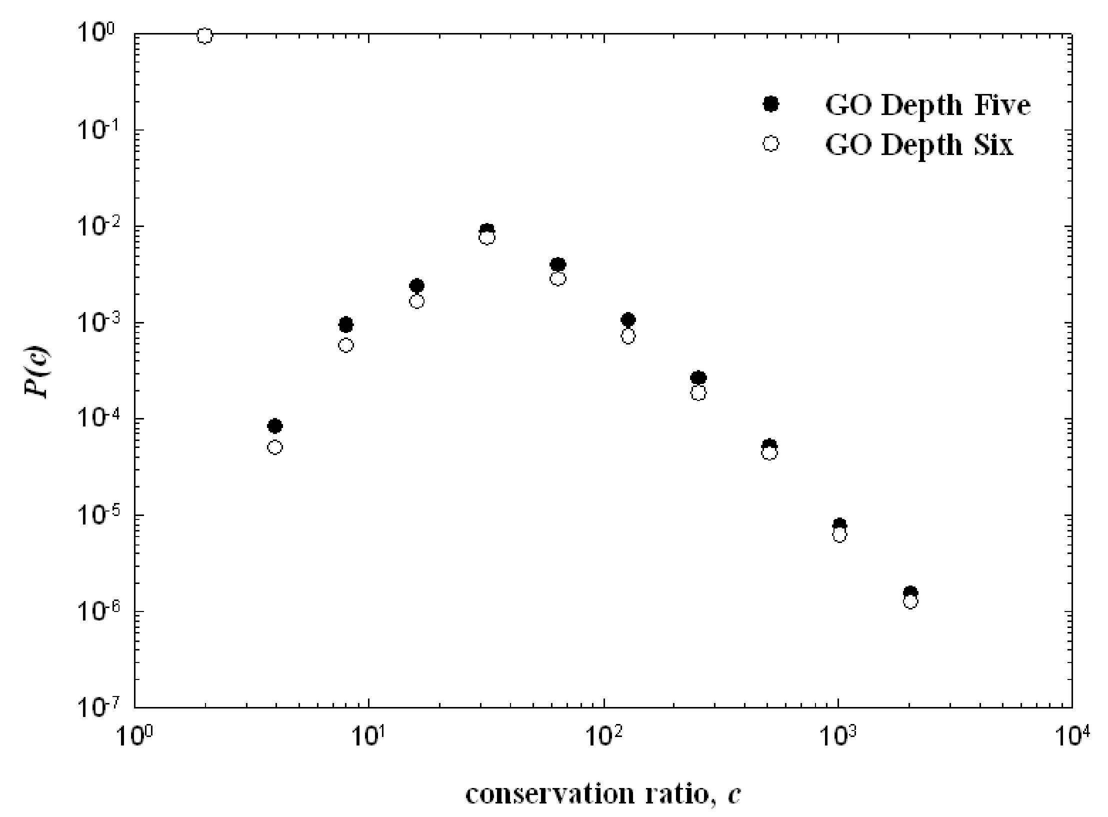

Motif topology: #4-1

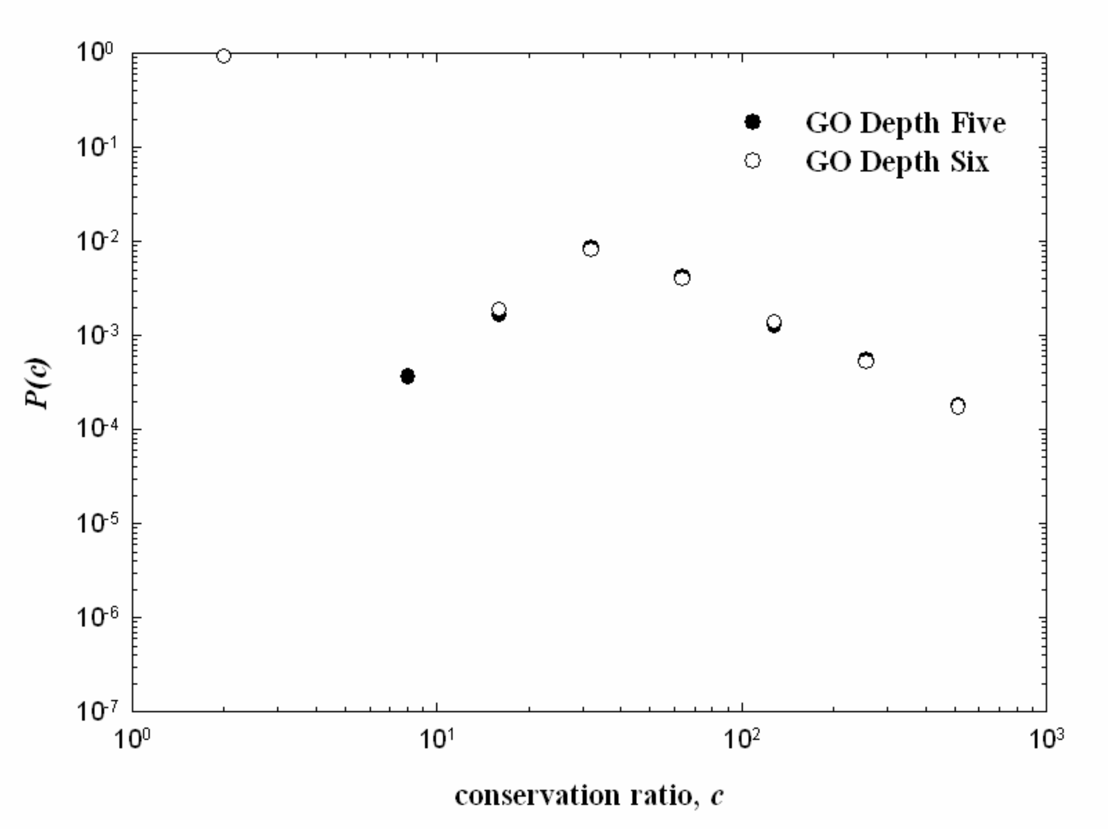

Motif topology: #4-2

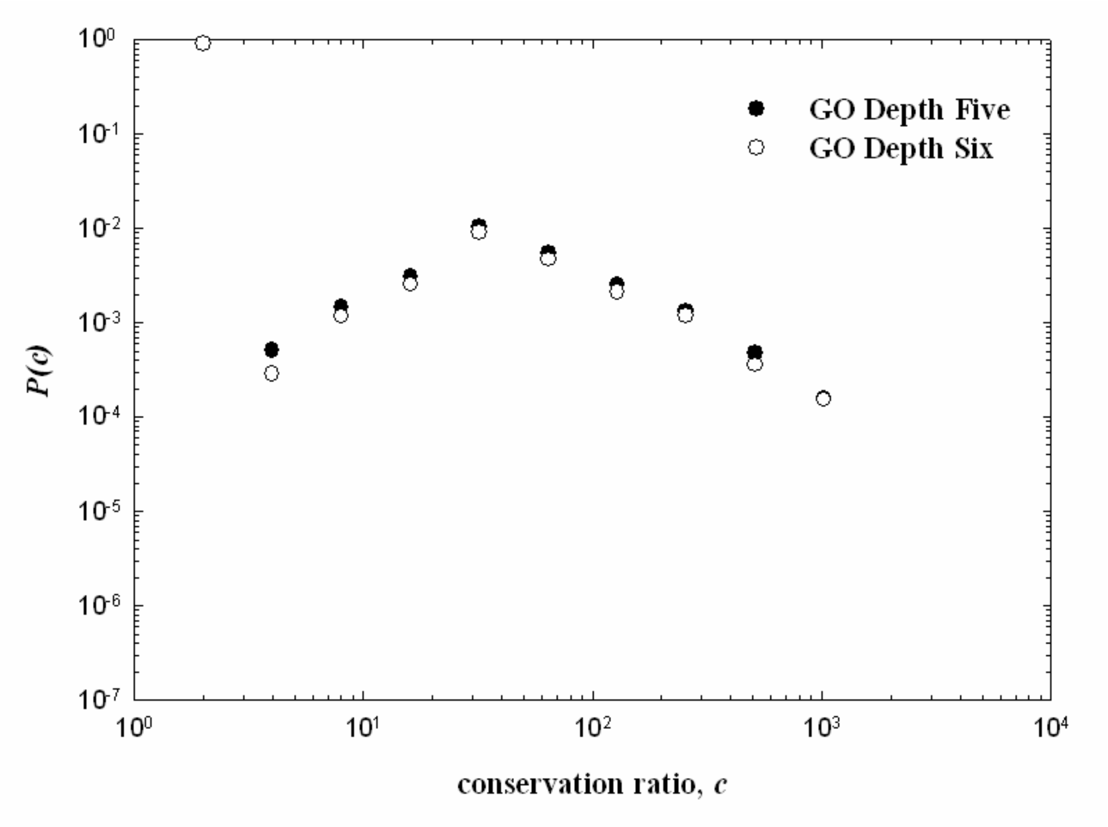

Motif topology: #4-3

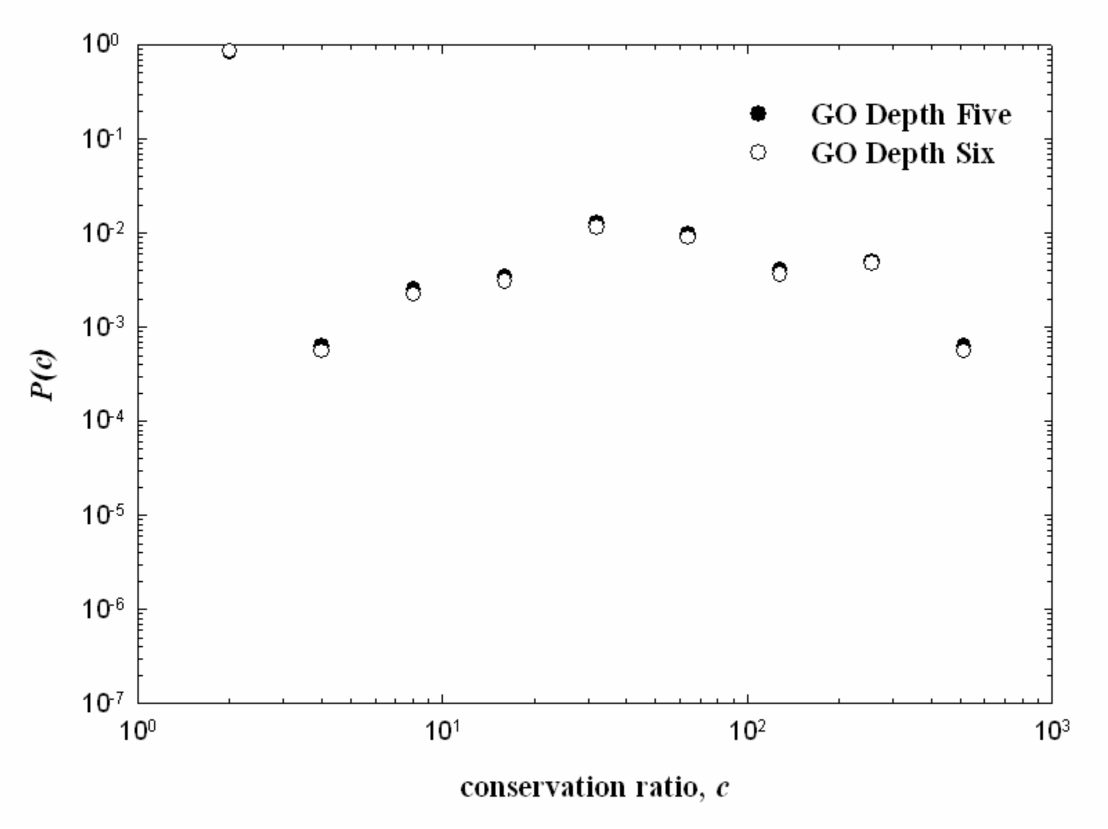

Motif topology: #4-4

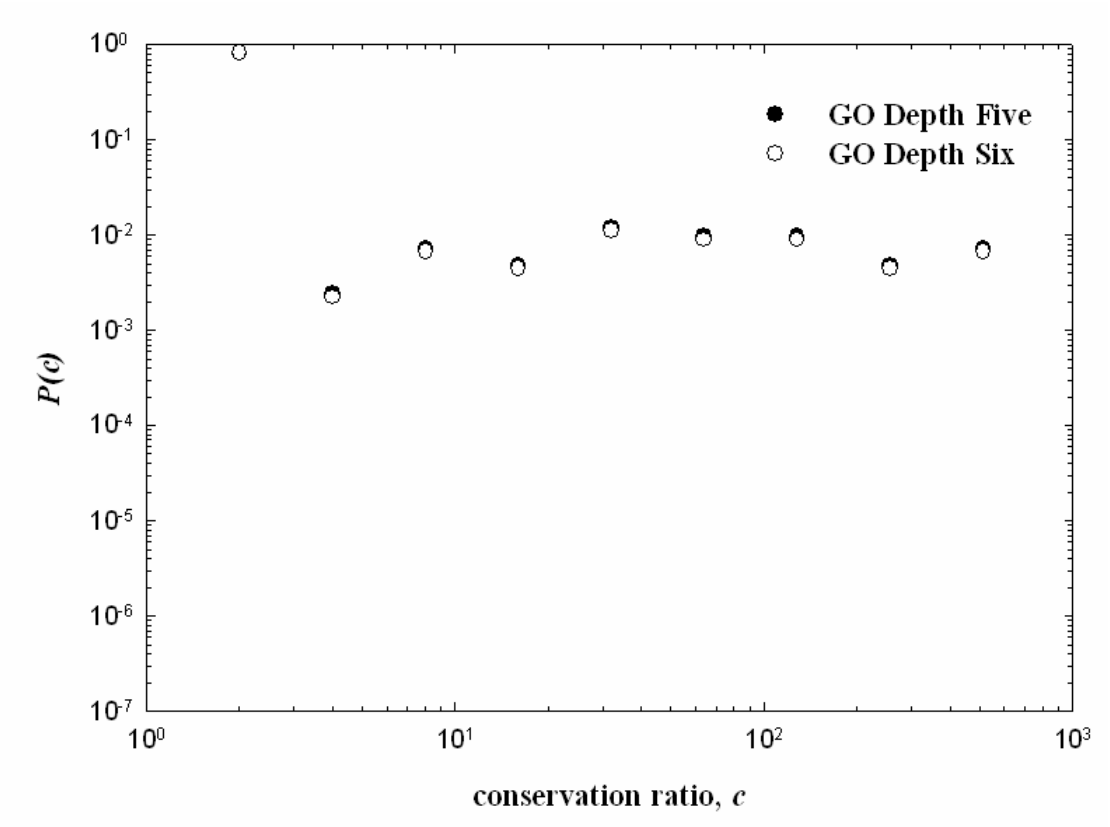

Motif topology: #4-5
